# An Efficient Communication Analysis of Modal Typology

**DOI:** 10.1162/OPMI.a.313

**Published:** 2026-01-15

**Authors:** Nathaniel Imel, Qingxia Guo, Shane Steinert-Threlkeld

**Affiliations:** Department of Psychology, New York University, New York, NY, USA; Department of Linguistics, University of Washington, Seattle, WA, USA

**Keywords:** modals, typology, semantic universals, efficient communication

## Abstract

The meanings expressed by the world’s languages have been argued to support efficient communication. Across diverse semantic domains, crosslinguistic analyses show that natural language vocabularies are jointly optimized for two competing pressures: cognitive simplicity and informative communication. This paper applies an efficiency analysis to modals (e.g., *can*, *ought*, *might*), a central mechanism for talking about situations other than the here-and-now. We define and measure the simplicity and informativeness of a large number of logically possible modal systems, together with a sample of twenty-seven natural language inventories. We also consider a recently-introduced semantic universal for modal expressions in natural language, dubbed the Independence of Force and Flavor (IFF). Our analysis yields three main results: (i) every optimal modal system perfectly satisfies the IFF universal; (ii) as systems contain more IFF modals, they become more efficient; (iii) attested modal systems are more efficient than merely possible systems. These results indicate that general pressures for efficient communication can explain typological variation in the lexicalization of modality.

## INTRODUCTION

The languages of the world exhibit *constrained variation*. While they differ substantially in important ways, there are also many possible but unattested languages, and actual languages exhibit considerable shared structure. Put differently, only a small subset of the mathematically possible languages have ever been spoken by any linguistic community. One goal of theoretical linguistics consists in accurately characterizing and explaining this subset, i.e., in identifying the ‘humanly possible’ languages. Such explanations can occur at every level of linguistic analysis. The field of *semantic typology* asks: which meanings are attested cross-linguistically, and why (Bach & Chao, 2009; Kemp et al., 2018)?

In many domains, robust constraints on the meanings expressed in the languages of the world—*semantic universals*—have been discovered (Bach & Chao, 2009; Barwise & Cooper, 1981; von Fintel & Matthewson, 2008). When such universals are found, it is natural to want to explain them as well. An idea with roots in the functionalist tradition of linguistics proposes that the meanings observed across languages are shaped by a pressure for efficient communication (Kemp et al., 2018; Zipf, 1949).

This paper evaluates this efficiency hypothesis in terms of the trade-off between cognitive simplicity and communicative informativeness (Kemp & Regier, 2012; Kemp et al., 2018; Levinson, 2012), in the domain of *modals*, from the perspective both of semantic universals and typology more broadly. A modal is typically considered to be a semantic operator that qualifies the truth of an expression. In English, these can be expressed by auxiliaries including *might*, *may*, *must*, *could* and adverbs like *probably*, *necessarily* among a variety of other constructions. Cross-linguistically, these meanings are expressed by diverse lexical categories and strategies (Bhatt et al., 2011; Cable, 2017).

Modality exemplifies the property that Hockett (1960) named displacement: the phenomenon of talking about possibilities beyond the actual here-and-now. Modals are typically also context-sensitive: words like *can* and *must* do not fully specify what kind of modality (i.e., relevant facts about knowledge, norms, or ability) a speaker has in mind, which means that listeners must rely on context to disambiguate the intended sense of possibility (see Modality section). In this paper, we explore whether efficiency can explain *why* modals lexicalize the specific combinations of possibility and necessity that are crosslinguistically attested. In other words, we are interested in whether efficiency can explain why words like *can* and *must* mean what they actually mean.

To evaluate the efficient communication hypothesis, we measure the complexity and informativeness achieved by a sample of 27 natural language modal systems, comparing them to a large number of hypothetical modal systems. We also consider a lexeme-level semantic universal that has been proposed to account for robust constraints on crosslinguistic variation in modal semantic systems, the Independence of Force and Flavor (IFF; Steinert-Threlkeld et al., 2023). We observe that (i) every optimal modal system consists only of IFF modals; (ii) as systems contain more IFF modals, they become more efficient; (iii) attested modal systems are more efficient than merely possible systems. Together, these results suggest that communicative efficiency can provide a robust explanation both of the presence of a semantic universal and broader patterns in semantic typology in the domain of modals.

The paper is structured as follows. We first provide an overview of cross-linguistic semantic variation for modals (Modality section). We explain the IFF semantic universal in Modal Semantic Universals section. We then introduce the simplicity/informativeness trade-off and describe in detail how to measure these properties in the modal domain (Efficient Communication as an Approach to Explaining Semantic Typology section). The main computational experiment and results are presented in Computational Experiment section. After showing that all of the main results are robust to various alternative ways of measuring informativeness and complexity (Robustness to Alternative Notions of Complexity and Informativeness section), we conclude with a discusssion of modeling decisions and areas for future work (Discussion section).

## MODALITY

Modals are expressions that are used to talk about alternative ways the world could be, over and above the way the world actually is. Paradigms are certain English auxiliaries like *may* and *must*. Since at least Kratzer (1981), the semantics of modals have been explicated in terms of two axes of variation: **force** and **flavor**. These axes can be illustrated with the following examples.

(1)  a. [Context: a friend walks in and shakes off a wet umbrella. You say:]
      It *must* be raining.
    b. [Context: you are reading the specifications of a homework assignment. It partially reads:]
      You *must* upload your homework as a PDF.
(2)  a. [Context: a friend is leaving and grabs an umbrella on the way out, saying:]
      It *may* be raining.
    b. [Context: a mother offers a treat to a child for finishing an assignment:]
      You *may* have a cookie.

The *must* examples exhibit strong (i.e., universal) force, but differ in flavor. For example, (1a) can be glossed as saying: all of the worlds compatible with my evidence are worlds in which it is raining. The universal quantification represents the force, and the domain of worlds (those compatible with my evidence) the flavor, in this case epistemic. (1b) exhibits universal force with deontic flavor, roughly saying that all the worlds in which you follow the rules are ones in which you upload a PDF. The examples with *may* in (2) exhibit weak (i.e., possibility) force: their meaning says that some world satisfies the prejacent. (2a) and (2b) again differ in flavor, with the former being epistemic and the latter being deontic.

In addition to epistemic and deontic flavors, many others have been identified: bouletic (worlds in which desires are fulfilled), teleological (worlds in which goals are satisfied), et cetera. Similarly, there are arguably more forces than just weak and strong: for instance, there are weak necessity modals (e.g., *should*, *ought*) which intuitively express universal quantification over a smaller domain of worlds (von Fintel & Iatridou, 2008). See Matthewson (2016) and references therein for further discussion of these two axes.

The examples above show that English modals lexically specify modal force (each modal has a fixed quantificational force) but exhibit variability across flavors (the modals can express more than one flavor). We note that such variability does not require that all modals in English can express all flavors: for instance, *might* arguably can only be used epistemically. Kratzerian semantics for modals capture this by hard-coding quantificational force into the meaning of a modal but relying on context to determine the flavor.^1^

Not all languages are like English: some exhibit so-called *variable force modals*, which specify flavor but do not lexically encode force. This has been found at least in St’át’imcets (Rullmann et al., 2008), Nez Perce (Deal, 2011), Old English (Yanovich, 2016), and Pintupi-Luritja (Gray, 2021).^2^ We illustrate the phenomenon with elicited examples of St’át’imcets *k’a* from Rullmann et al. (2008):^3^

(3)  a. [Context: You have a headache that won’t go away, so you go to the doctor. All the tests show negative. There is nothing wrong, so it must just be tension.]
      nilh *k’a* lh(el)-(t)-en-s-wá(7)-(a)    ptinus-em-sút
      foc infer from-det-1sg.poss-nom-impf-det think-mid-ooc
       ‘It *must* be from my worrying.’
    b. [Context: His car isn’t there.]
      plan k’a qwatsáts
      already infer leave
      ‘Maybe he’s already gone.’

(3a) shows *k’a* being used with strong force and epistemic flavor. (3b) shows *k’a* being used with weak force and epistemic flavor. Further analysis in Rullmann et al. (2008) shows that *k’a* can only be used with epistemic flavor, so it is an example with lexically specified flavor but variable force. The discussed semantic variation across English and St’át’imcets is summarized by Table 1.

**Table 1.** Two kinds of modal semantic underspecification: *variable-force* and *variable-flavor*.

St’át’imcets *k’a*				English *must*			
	epistemic	deontic	…		epistemic	deontic	…
weak	✓			weak			
strong	✓			strong	✓	✓	

Even with this substantial cross-linguistic variation, there are kinds of modal meanings that are unnattested. We can describe potential restrictions on modal meanings as semantic universals. In order to state universals for modals in a relatively theory-neutral manner (i.e., in a way that does not presuppose a particular formal semantic implementation), we make the following assumptions. We assume that force and flavor are fundamentally properties of contexts of use. This reflects current practice in semantic fieldwork as applied to modality (Bochnak & Matthewson, 2020; Matthewson, 2004; Vander Klok, 2021).^4^ For example, the modal questionnaire of Vander Klok (2021) consists exactly of discourse contexts designed to isolate a single force-flavor pair. These contexts can subsequently be used for tasks like elicitation, translation, and acceptability. Finally, we will say that a modal *M can express* a force-flavor pair just in case a bare positive sentence of the form *Mp* is judged felicitous in a context with that pair.^5^

At this level of generality, we will represent the meaning of a modal as being a set of force-flavor pairs. The semantic universals that we will discuss will be constraints on what kinds of meanings (sets of such pairs) are attested in the languages of the world. For notation, for a modal *m*, let 〚*m*〛 be the set of force-flavor pairs it can express. Furthermore, we will write fo(*m*) = {fo | ∃fl s.t. (fo, fl) ∈ 〚*m*〛} for the set of forces that a modal *m* can express and *mutatis mutandis* for fl(*m*) and the set of flavors.

We adopt this level of generality because it avoids commitment on the exact formal semantics of these expressions, which is often still being debated. For example, we can say that a *variable force modal* is one that can express more than one pair with the same flavor (i.e., for which |fo(*m*)| > 1). This is useful because there are two broad approaches to the semantics of such variable force modals: they actually encode existential quantification but lack an explicit universal counterpart in the language (Deal, 2011) or they encode universal quantification but rely on some mechanism of domain restriction (Bochnak, 2015a; Močnik & Abramovitz, 2019; Rullmann et al., 2008). On such analyses, the underlying semantics contains one specific quantifier; in the present setting, they will still be considered variable force since bare positive sentences are used in contexts with multiple forces.

## MODAL SEMANTIC UNIVERSALS

Having laid this groundwork, we now introduce the Independence of Force and Flavor (IFF) universal (Steinert-Threlkeld et al., 2023) by showing how it is a refinement of an earlier proposed universal which accommodates recently-discovered counterexamples.

### The Single-Axis of Variability Universal

While the previous section has shown that some modals exhibit variability on the flavor axis (e.g., English *may*) and some modals exhibit variability on the force axis (e.g., St’át’imcets *k’a*), all of the previously discussed expressions are not variable on the other axis. This pattern was observed across many languages from many different families. As a result of a detailed study of the modal systems of six typologically unrelated languages, Nauze (2008) proposed a semantic universal stating that modals cross-linguistically can in fact only exhibit variation along a single axis:

The Single Axis of Variability (SAV) Universal: All modals in natural language satisfy the single axis of variability property: if a modal can express more than one flavor, it can only express one force (and *mutatis mutandis* for force and flavor). That is to say: a modal may exhibit variable force or variable flavor, but not both.^6^

[Alternative formulation: |fo(*m*)| = 1 or |fl(*m*)| = 1, where |·| is the set cardinality function.]

At least two counterexamples to this universal have been discovered. The first comes from Washo. Bochnak (2015a, 2015b) has argued that the modal verb *-eʔ* can be used in both possibility and necessity contexts with a range of modal flavors. In other words, it exhibits variation both on the force axis as well as the flavor axis. Similarly, Močnik and Abramovitz (2019) demonstrate that the Koryak attitude verb *ivək* can be used to express both necessity and possibility. For the doxastic flavor, this means that *ivək* can be used to mean roughly ‘believe’ (necessity) as well as ‘allow for the possibility that’ (possibility). They also argue that the expression can be used to express both doxastic and assertive flavors, thus demonstrating variability on both axes.^7^ It is worth noting that while *ivək* exhibits variability along both the force and flavor axes, it is not maximally underspecified: there are still force-flavor combinations that it *cannot* express. Bochnak and Močnik et al. use different variants of the universal quantifier plus choice function analysis of Rullmann et al. (2008) to analyze the respective expressions.

We note also that a refinement of Nauze’s SAV due to Vander Klok (2013b) (as reported and discussed in Matthewson, 2016) does not accommodate these counterexamples. In particular, Vander Klok proposes that *a modal system as a whole* may only exhibit variability on a single axis in each of the root and epistemic domains. That is: if one root modal exhibits variability on the flavor axis, no other root modal exhibits variability on the force axis (though an epistemic modal may do so) and *mutatis mutandis* for epistemic modals and also for the force axis. This proposal is strictly stronger than Nauze’s: if a language satisfies Vander Klok’s generalization, then every modal therein satisfies SAV. For this reason, counterexamples to the SAV are also counterexamples to this proposal.

### The Independence of Force and Flavor Universal

The counterexamples to the SAV universal show that some languages have modals which are contextually underspecified for *both* force and flavor. It does not follow from this, however, that arbitrary sets of force-flavor pairs are expressed. Intuitively, one does not expect to find a modal in a language that can only express, for instance, epistemic necessity and teleological possibility. Steinert-Threlkeld et al. (2023) use this intuition to define a new semantic universal for modals.

The Independence of Force and Flavor (IFF) Universal: All modals in natural language satisfy the independence of force and flavor property: if a modal can express the pairs (fo_1_, fl_1_) and (fo_2_, fl_2_), then it can also express (fo_1_, fl_2_) and (fo_2_, fl_1_).

[Alternative formulation: a modal *m* satisfies the IFF property just in case 〚*m*〛 = fo(*m*) × fl(*m*), where × is the Cartesian product.]

This universal captures the guiding idea from Kratzer (1981) and much subsequent theorizing on the semantics of modals that force and flavor are *independent* axes of meaning. This standard semantics embodies this independence by separating quantificational force from the domain (of possible worlds) over which the quantifier ranges. Roughly: modals are either existential or universal quantifiers, but two contextual parameters—the modal base and ordering source—jointly determine the range of worlds, and thereby the flavor, quantified over. For example, ‘must’ is a universal quantifier; in a deontic use, the worlds it ranges over are worlds consistent with a given set of rules, while an epistemic use could range over worlds consistent with a given body of evidence. The IFF universal expresses this conception of independence in a theory-neutral way and proposes it as a substantive universal on the semantics of modals cross-linguistically.^8^

One can next ask: why might the IFF universal be true? A growing body work has explored domain-general explanations of semantic universals, including accounts from *ease of learning* and *communicative efficiency* (Denić et al., 2022; Steinert-Threlkeld, 2020, 2021; Steinert-Threlkeld & Szymanik, 2019, 2020; Uegaki, 2023; van de Pol et al., 2021; Zaslavsky et al., 2018: i.a.). In this work, we explore whether the IFF universal may have emerged due to the latter; that is, we investigate whether optimally trading off cognitive complexity with communicative accuracy tends to generate languages that more often satisfy IFF.^9^ Our aim is thus to illuminate not only whether actually attested modal systems are shaped by efficiency, but also whether there is a systematic relationship between efficiency and ‘naturalness’.

## EFFICIENT COMMUNICATION AS AN APPROACH TO EXPLAINING SEMANTIC TYPOLOGY

Our working notion of communicative efficiency can be summarized by the following tension. A language can be simple and uninformative (e.g., containing a single expression). A language can be complex and informative (e.g., containing unique expressions for each possible thought to be expressed). A language cannot be both simple and informative: these two pressures trade-off against each other. A popular idea in linguistics and cognitive science is that the natural languages are (near) solutions to this multi-objective optimization problem, and that these efficiency pressures explain constraints on crosslinguistic variation (Kemp et al., 2018).

This efficient communication approach has seen empirical success across a variety of semantic domains including kinship terms, color terms, number terms, container terms, quantifiers, boolean connectives, indefinite pronouns and deictic adverbs, among others (Chen et al., 2023; Denić et al., 2022; Kemp & Regier, 2012; Regier et al., 2015; Steinert-Threlkeld, 2021; Uegaki, 2023; Xu & Regier 2014; Xu et al., 2016; Zaslavsky et al., 2018, 2021). We follow others in this literature by using computational modeling to evaluate whether actual human languages are optimized for efficiency, over and above the many mathematically possible languages.

To show that the natural language modal inventories and the semantic universals that hold of them are consistent with having been shaped by general pressure for efficient communication, we require measures of complexity and informativeness that are appropriate to modal semantics. In the remainder of this section, we describe our measures of simplicity and informativeness in detail. Henceforth, we will use the term ‘language’ to mean a modal inventory, i.e., a multiset of modals and their meanings.^10^

### Simplicity

We define simplicity in terms of its inverse, complexity. Following Feldman (2000), who showed that the learnability of Boolean concepts is well-predicted by their length in literals (i.e., number of positive or negative mentions of a variable), we model the complexity of a modal meaning as the smallest number of atoms it takes to express its meaning in a domain-specific representation language, or Language of Thought (LoT) (Denić et al., 2022; Feldman, 2000; Fodor, 1975; Goodman et al., 2008; Kemp & Regier, 2012; Piantadosi et al., 2016) for modals. This representation language is the standard language of propositional logic. The language has an atom both for each flavor and for each force. The primitive operators in the language include conjunction (∧), disjunction (∨), and negation (¬) of features. As an example, in this language, we can express the meaning of English *might*, 〚*might*〛 = {(weak, epistemic)} as *w* ∧ *e*, where *w* is the atom for weak force and *e* is the atom for epistemic flavor.

We obtain the minimum description length expressions for all the meanings in our semantic space using a boolean grammar, generating and sorting all possible formulae up to a given maximum length. This procedure is guaranteed to find the shortest formula for each modal meaning. This allows us to map any modal meaning to a discrete measure of its complexity, using a collection of the points it can express. One important fact about our modal LoT is that conjunction distributes over disjunction, allowing one to replace a formula like (*w* ∧ *e*) ∨ (*w* ∧ *d*) with a formula like *w* ∧ (*e* ∨ *d*). This reflects the intuition that some meanings differ in terms of in how difficult it is to compactly represent their variability on the two axes. In particular, when features of meaning share an axis, this axis may be ‘factored’ out in the shortest formula representation. Some example results of our algorithm are illustrated in Table 2.

**Table 2.** Measuring complexity for English *may* and two hypothetical modals *mought* and *notcirc*. First column: meaning representation. Here: *w* and *s* stand for weak and strong force, respectively, and *e*, *d*, *c*, and *t* for the flavors epistemic, deontic, circumstantial, and teleological. Second column: shortest LOT formula. Third column: complexity.

**Modal**	**Meaning representation**					**Shortest Formula in LOT**	**Complexity (# of atoms)**
*may*		*e*	*d*	*c*	*t*	*w* ∧ (*e* ∨ *d*)	3
	*w*	✓	✓				
	*s*						
*mought*		*e*	*d*	*c*	*t*	(*w* ∧ *e*) ∨ (*s* ∧ *d*)	4
	*w*	✓					
	*s*		✓				
*notcirc*		*e*	*d*	*c*	*t*	¬*c*	1
	*w*	✓	✓		✓		
	*s*	✓	✓		✓		

Given this measure of the complexity of any modal in isolation, we can measure the overall complexity of a language as a sum of the complexities of the modals therein. Formally:

$$\text{Comp}\left(L\right)≔\sum_{m\in L}\min\left\{\text{len}\left(\varphi \right):\varphi \in \text{LOT},\left[\varphi \right]=〚m〛\right\},$$ (1)

where *φ* is a (well-formed) formula generated by the boolean LoT grammar. For example, if a language consisted of exactly one of each of the modals in Table 2, it would be assigned Comp(*may*) + Comp(*mought*) + Comp(*notcirc*) = 8. To summarize, to quantify the complexity of modal languages we use a minimum description length approach and model the complexity of a language as a sum of the complexities of each of the items in its vocabulary.

### (Literal) Informativeness

The informativeness of a language is modeled after the idea of successful communication between literal speakers and listeners (Skyrms, 2010; Steinert-Threlkeld, 2021). This measure can be modeled as an expected utility of a language *L* for communication, where the expectation is taken over repeated interactions between a speaker trying to successfully convey a force-flavor pair *p* ∈ *P* to a listener. More precisely:

$$\begin{array}{l} I\left(L\right)≔𝔼\left[u\left(p,p^{\prime }\right)\right]\\ \;=\sum_{p\in P}\mathbb{P}\left(p\right)\sum_{m\in L}\mathbb{P}\left(m|p\right)\sum_{p^{\prime }\in m}\mathbb{P}\left(p^{\prime }|m\right)\cdot u\left(p^{\prime },p\right) \end{array}$$ (2)

In Equation 2, ℙ(*m*|*p*) is the probability a speaker selects a specific modal *m* to communicate a meaning *p* (a single (fo, fl) pair in the semantic space). ℙ(*p*′|*m*) is the probability that a listener guesses a (fo, fl) pair *p*′, given the expression heard (*m*). We assume that ℙ(*m*|*p*) is uniform on expressions that can express *p*, and that ℙ(*p*′|*m*) is uniform on meaning points that *m* can express. A prior over meaning points ℙ(*p*) models how often agents need to communicate about specific meanings. In principle, different linguistic communities will have different communicative need distributions. We estimate one distribution from English corpus data (Pyatkin et al., 2021) to measure informativeness for every language in our main experiment (details in Methods section), leaving estimation of the communicative need distributions of the other languages to future work.

The utility function *u*(*p*, *p*′) measures how ‘good’ the listener’s guess *p*′ is, if the speaker intended to convey *p*. The structure present in the modal meaning space allows us to model communicative utility as a graded notion, with some utility awarded to guesses that are better than others. In particular, we define a utility scoring function *u*(*p*, *p*′) which gives half-credit (0.5) to correctly guessing each of the force and the flavor of *p*. Thus, if *p*′ shares one axis of meaning with *p*, the utility will be 0.5; if it shares both, 1; and if it shares neither, 0. More precisely:

$$u\left(p,p^{\prime }\right)=0.5\cdot 1\left\{\text{fo}\left(p\right)=\text{fo}\left(p^{\prime }\right)\right\}+0.5\cdot 1\left\{\text{fl}\left(p\right)=\text{fl}\left(p^{\prime }\right)\right\}$$ (3)

where **1**{*x*} is the indicator function which returns 1 if *x* is true, and 0 if *x* is false.

Lastly, just as we measure complexity instead of simplicity, we define the *communicative cost* of a language as the ‘inverse’ of its informativeness: *Cost*(*L*) = 1 − *I*(*L*). In other words, while simplicity and informativeness are “desirable” features for a language, complexity and communicative cost are “undesirable” features: they should both be *minimized* to the extent possible.

To summarize: our measure of informativeness makes concrete choices about (i) the prior over force-flavor pairs, (ii) the utility function, and (iii) the nature of the speaker and listener models (in particular, that they are so-called ‘literal’ agents who do not engage in any pragmatic reasoning). After presenting our primary results in the next section, we show in Robustness to Alternative Notions of Complexity and Informativeness section that these results are robust to making different choices in all of (i)–(iii).

## COMPUTATIONAL EXPERIMENT

In order to evaluate the simplicity/informativeness trade-off for modals, we will measure the *optimality* of a language as the distance to the optimal solutions along an estimated Pareto frontier. The Pareto frontier is a set of solutions to a trade-off problem where no improvement in one dimension is possible without getting worse along the other. In our setting, this corresponds to the set of languages that achieve the minimum complexity for a given value of communicative cost. We will also measure the degree to which each language satisfies IFF to see if this variable—which we will call *naturalness*—correlates with optimality. Accordingly, the experiment involves the following steps: we (1) collect a sample of natural language modal inventories to measure; (2) fix a semantic feature space from which to generate meanings; (3) find the shortest expression for each meaning; (4) estimate the communicative need distribution over meanings; (5) estimate the Pareto frontier; (6) generate a sample of hypothetical modal systems; and (7) measure the optimality of natural and hypothetical languages by each language’s distance to the frontier. We describe these steps in turn in the next section, before presenting our main results. The code for reproducing these results can be found at https://github.com/nathimel/modals-effcomm.

### Typological Data

To measure the communicative efficiency of natural language modal inventories, we use the recently introduced Database of Modal Typology (Guo et al., 2022). This is a public repository for linguists to contribute data they have collected on crosslinguistic modal semantics. Individual modal expressions are annotated for force and flavor, among other linguistic features. At time of writing, the database contains 40 languages; of these, we consider only the languages for which linguists indicated the modal inventories were described completely, with positive and negative truth value judgments from speakers in elicitation tasks. This restriction results in a total of 27 languages from 17 families that we use for our analysis, described in Table 3. This filtered sample also constrains the maximum language size and meaning space used in the experiment (more details in Methods section). A sample of the Tlingit data recorded in the database and used in our analyis is given in Table 4.

**Table 3.** The 27 languages used in our computational experiment, taken from the modal typological database described in Guo et al. (2022). The data for these languages result from published and unpublished elicitation tasks and span 17 distinct families.

Name	Family	Source
Akan	Atlantic-Congo	Uegaki et al. (2025)
Basque	Basque	Uegaki et al. (2025)
Cantonese	Sino-Tibetan	Uegaki et al. (2025)
Central Khmer	Austroasiatic	Uegaki et al. (2025)
Dutch	Indo-European	Uegaki et al. (2025)
Gitksan	Tsimshian	Matthewson (2013)
Goemai	Afro-Asiatic	Hellwig (2011)
Hausa	Afro-Asiatic	Uegaki et al. (2025)
Modern Hebrew	Afro-Asiatic	Uegaki et al. (2025)
Hindi	Indo-European	Uegaki et al. (2025)
Hungarian	Uralic	Uegaki et al. (2025)
Javanese	Austronesian	Vander Klok (2013a)
Korean	Koreanic	Uegaki et al. (2025)
Lillooet	Salishan	Rullmann et al. (2008)
Logoori	Atlantic-Congo	Gluckman and Bowler (2020)
Mandarin	Sino-Tibetan	Uegaki et al. (2025)
Modern Greek	Indo-European	Uegaki et al. (2025)
Tundra Nenets	Uralic	Nikolaeva (2014)
Turkish	Turkic	Uegaki et al. (2025)
Igbo	Atlantic-Congo	Uegaki et al. (2025)
Japanese	Japonic	Uegaki et al. (2025)
Russian	Indo-European	Uegaki et al. (2025)
Tagalog	Austronesian	Uegaki et al. (2025)
Tharaka	Atlantic-Congo	Uegaki et al. (2025)
Tlingit	Athabaskan-Eyak-Tlingit	Cable (2017)
Vietnamese	Austroasiatic	Uegaki et al. (2025)
Western Farsi	Indo-European	Uegaki et al. (2025)

**Table 4.** Example of our basic data format for several strategies of expressing modality in Tlingit (Cable, 2017).

expression	force	flavor	can_express
giwe	weak	circumstantial	0
shákdé	weak	circumstantial	0
future mode	weak	circumstantial	0
potential mode	weak	circumstantial	1

All the languages we measure in this work are represented as a collection of their modal expressions, and in turn each expression is represented as a collection of the force-flavor pairs it can be used to communicate. The natural languages are obtained from the database, while the hypothetical ones are mathematically generated. We describe this generation procedure in the next subsection.

### Methods

#### Meaning Space.

Our main results center a meaning space *P* with two modal forces {weak; strong} and three modal flavors {epistemic, deontic, circumstantial}, for a total of six possible meaning points. This meaning space was chosen because it is the maximal set of force-flavor pairs that could be expressed by the natural language modal inventories (i.e., the intersection of the languages’ observed meanings).^11^ An individual modal’s meaning will be modeled as a subset of these six points. The possibility of modeling languages with different ‘domains’ of modality (possibly hierarchically structured with, e.g., root/epistemic as being fundamental) will be left for future work.

#### Shortest Expressions.

There are 2^6^ − 1 = 63 modal meanings in this space (non-empty sets of force/flavor pairs). For each of these, we use the enumeration algorithm described in Simplicity section to find the shortest formula expressing that meaning, thereby determining the complexity of each modal meaning.

#### Communicative Need Distribution.

We estimate one communicative need distribution over force-flavor pairs to measure the informativeness of all languages in the experiment. This is done using a fine-grained annotation of the Georgetown Gradable Modal Expressions (GME) Corpus, as described in Pyatkin et al. (2021). The GME is an expert-annotated sample of documents included in the Opinion Corpus introduced by Wiebe et al. (2005), which consists of roughly ten thousand sentences drawn from articles in English from the world press published during 2001 and 2002. We use the “Fine-Grained” annotation category of data from Pyatkin et al. (2021) which uses a taxonomy of modal flavors compatible with our chosen feature space. The communicative need distribution is the relative frequencies of each force-flavor pair observed in the corpus. To obtain these frequencies, we count the occurrences of modal verbal auxiliaries, which were annotated for flavor (and modal force is unambiguous in English). After dropping observations of modal flavors that weren’t part of our set (such as ‘agentive,’ for example), we normalized the remaining counts to obtain the relative frequencies for each force-flavor pair. The resulting distribution is displayed in Table 5.

**Table 5.** Estimated probability distribution over force-flavor pairs, representing a communicative need distribution over modal meanings.

	epistemic	deontic	circumstantial
weak	0.139	0.042	0.143
strong	0.104	0.254	0.318

#### Estimating the Pareto Frontier.

To estimate the Pareto frontier of languages that optimally balance complexity and communicative cost, we apply an evolutionary algorithm to directly optimize these two objectives (Denić et al., 2022; Steinert-Threlkeld, 2019, 2021). This works as follows. In the beginning, a seed population of 2000 artificial modal languages is randomly generated (using the first sampling procedure described in the next section). There are then multiple (200) ‘generations’. At the end of each generation, a random choice of between 1 and 5 mutations is applied to each of the dominant languages. These dominant languages represent the subset of their generation best optimizing the simplicity/informativeness trade-off. The mutations include randomly adding a modal to a language, removing a modal from a language, and replacing a modal in a language. Another mutation removes a single force/flavor pair from the meaning of a given modal in a language, and the last mutation adds to a language one modal that can express only a single force/flavor pair. Each dominant language has enough ‘offspring’ via mutation to create 2000 languages at each generation. After 200 generations of this process, the dominant languages are the estimated Pareto frontier.^12^

#### Sampling Languages.

In addition to measuring the modal inventories of natural languages, we also generate a large sample of hypothetical modal inventories. Because exhaustive enumeration of the space of possible modal languages is not feasible,^13^ we use several sampling techniques to encourage exploration of the space of possible languages.

Our sampling procedure has two steps. First, one sample of languages is obtained from random/unbiased sampling. We manipulate both the size of the language (from one to ten modals) and the number of modals in the language satisfying the IFF universal (from one to the current size). The maximum vocabulary size was chosen for feasibility reasons, but also because the largest vocabulary in our typological data contains ten modals. For each combination of these two parameters, we generate languages by sampling mutisets of modal expressions from the set of possible expressions that do and do not satisfy IFF.

Second, to encourage significant exploration of the space of possible languages, especially the low-density regions unlikely to be discovered by the above random sampling procedure, we apply the same evolutionary algorithm for estimating the Pareto frontier of efficient languages three more times: once for each of the other corners of the two-dimensional (complexity, communicative cost) space of possible languages. In other words, while the main evolutionary algorithm sought to *minimize* both measures, in order to encourage exploration, we look at all combinations of minimizing and maximizing both measures.

We combine all the unique languages discovered in the experiment, (i) by random sampling and (ii) each of the four runs of the evolutionary algorithm, into one pool of languages. Including the 27 natural languages, we obtain a sample of 32301 total languages for our analysis.

#### Measuring Optimality.

Finally, each language is measured for complexity, communicative cost, and *optimality*. We define the optimality of a language to be its (inverse) minimum Euclidean distance to a point on the Pareto frontier.^14^

#### Hypotheses.

We measure *naturalness* as a continuous value of languages (the fraction of the vocabulary conforming to the IFF universal). If the modal inventories in natural language have been shaped by the simplicity/informativeness trade-off, then we expect that the natural languages will be among the optimal languages, and that optimality will be significantly correlated with the IFF degree.

### Results

**Figure 1.**
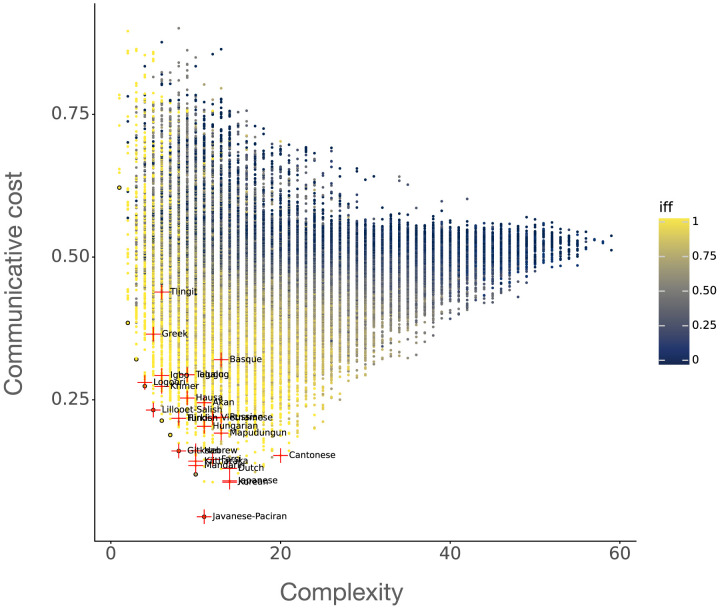
The complexity/communicative cost trade-off for modal languages. The black circles constitute the estimated Pareto frontier of optimal solutions to the trade-off. Red ‘+’ are natural languages obtained from Guo et al. (2022) and are labeled. The color of a language is its degree of naturalness (proportion of modals satisfying the IFF universal).

Figure 1 depicts the main results. This plot shows all of the *N* = 32301 languages generated as described above on a two-dimensional plane, with the *x*-axis being complexity and the *y*-axis being communicative cost. The black circles are the languages discovered during our search procedure that balance complexity and communicative cost better than any other languages. These languages constitute the estimated Pareto frontier. Each natural language is labeled and marked by a red ‘+’. The color corresponds to the naturalness, i.e., what percentage of the modals in the language satisfy IFF (with 0.0 being blue and 1.0 being yellow).

We catalogue three main results. First, all Pareto-optimal languages are ‘perfectly natural’. That is, every estimated solution to the simplicity/informativeness trade-off is a lexicon containing only modals that satisfy the IFF universal.

This leads to our second main observation: naturalness is highly correlated with simplicity, informativeness, and Pareto-optimality. The strength of some of these relationships can be seen observed from visual inspection: as languages become ‘more natural’ / ‘more IFF’, they also become simpler, more informative, and closer to the Pareto frontier. Measuring Pearson correlations shows that a language’s degree of naturalness is strongly correlated with both simplicity (*r*(*N* − 2) = .484, *p* ≈ 0), informativeness (*r*(*N* − 2) = .576, *p* ≈ 0), and with optimality (*r*(*N* − 2) = .537, *p* ≈ 0).^15^

Finally, our third observation regards the efficiency of actual modal languages. The 27 attested languages are closer on average to the Pareto frontier than merely mathematically possible, artificially generated languages. Even the languages that are further from the frontier (e.g., Tlingit, Basque, Cantonese, Mandarin, Japanese, Farsi, Mapudungun) appear to be significantly more efficient than many hypothetically possible languages. We return to a discussion of these languages in Discussion section. A comparison of mean simplicity, informativeness and Pareto optimality for the natural languages and the generated hypothetical languages is given in Table 6. This table shows that the natural languages are simpler, more informative, and also more optimal than merely possible languages.^16^

**Table 6.** Mean simplicity, informativeness, and Pareto optimality for attested natural languages vs. hypothetical languages.

language	*N*	simplicity	informativeness	optimality
natural	27	.790	.786	.937
population	32301	.647	.536	.776

Before moving forward to a general discussion of these results, we first ask: how well preserved are these trends under different ways of measuring efficiency? In the next subsection, we address this question by exploring the robustness of the main results to different modeling assumptions.

## ROBUSTNESS TO ALTERNATIVE NOTIONS OF COMPLEXITY AND INFORMATIVENESS

To ensure the generality and stability of our findings, we conducted a comprehensive series of robustness checks. These checks involved systematically varying key components of our communicative efficiency model. We first explored the impact of different parameterizations within our existing informativity measure, holding complexity constant. Subsequently, we compared our primary model against established alternative theoretical formalizations for both informativeness and complexity. Across these analyses, we found that our core conclusions can be recovered under some, but not all alternative notions of efficiency.

### Parameter Variations on Informativeness

First, we explored different parameterizations of our formulation of informativeness. Our informativeness measure, which is based on expected communicative utility, has three key components: the communicative need distribution, the speaker/listener distributions, and the communicative utility function. In what follows, we define alternative choices for each of these components.

#### Communicative Need: Estimated vs. Uniform.

Our main result was obtained using a prior over force-flavor meaning pairs that was estimated from a corpus. Since this estimate comes from one corpus within a single genre, it is unclear how representative it is as an accurate model of communicative need. To further explore the robustness of our results, we will also repeat our analysis using a uniform distribution. Future work may explore other ways of estimating communicative need in this domain (Zaslavsky et al., 2020), including estimating different distributions on a per-language basis.

#### Communicative Utility: Graded vs. Binary.

While our first experiment reported results using a graded notion of utility for modal-meanings (Equation 3), there are other ways of modeling a successful communicative interaction. Another reasonable criterion might require that only perfect transmission of a force-flavor pair should be counted as success. To this end, we define an additional utility function *u*_bin_(*p*, *p*′) which awards success for a communicative interaction if and only if *p* = *p*′. We then have *u*_bin_ simply be an indicator function:

$$u_{\text{bin}}\left(p,p^{\prime }\right)=1\left\{p=p^{\prime }\right\}.$$

#### Literal vs. Pragmatic Speakers.

So far, we have described how to measure the communicative utility of modal lexicons with respect to *literal* speakers and listeners. We now describe a measure of informative communication relative to *pragmatic* speakers and listeners, using models from the Rational Speech Act (RSA) framework (Degen, 2023; Frank & Goodman, 2012). The basic intuition for the RSA model is as follows. While a literal speaker does not weigh the benefits or costs of using particular expressions to communicate their intended meaning, a pragmatic speaker considers the utility of different expressions depending on the way a literal listener will interpret their utterance.^17^ A pragmatic listener chooses to interpret a modal expression based on how a pragmatic speaker would choose to utter the expression. These models are formalized in Equations 4 and 5:

$${\mathbb{P}}_{\text{prag}-\text{speaker}}\left(m|p\right)=\frac{\exp\left(\alpha \cdot U_{\text{prag}}\left(m,p\right)\right)}{\sum_{m^{\prime }\in M}\exp\left(\alpha \cdot U_{\text{prag}}\left(m^{\prime },p\right)\right)}$$ (4)

$${\mathbb{P}}_{\text{prag}-\text{listener}}\left(p|m\right)=\frac{\mathbb{P}\left(m|p\right)\cdot \mathbb{P}\left(p\right)}{\sum_{p^{\prime }\in M}\mathbb{P}\left(m|p^{\prime }\right)},$$ (5)

where the utility for a pragmatic speaker to choose a modal expression *m* to communicate a force-flavor pair *p* is defined in Equation 6. This utility is the log probability of the literal listener’s interpreting modal *m* as meaning *p*:

$$U_{\text{prag}}\left(m,p\right)=\log\mathbb{P}\left(p|m\right).$$ (6)

The idea behind this pragmatic assignment of utility is that the more likely that an expression will be interpreted correctly by a listener, the better it is for a speaker to use it.^18^ The pragmatic speaker chooses modal expressions in proportion to their utility.^19^ Meanwhile, the pragmatic listener selects the Bayesian-optimal force-flavor interpretation, given their knowledge about what modals mean in their language, and a prior over force-flavor pairs. With these ingredients in place, we now define the *pragmatic informativeness* of a language *L* in Equation 7:

$$I_{\text{prag}}\left(L\right)=\sum_{p\in M}\mathbb{P}\left(p\right)\sum_{m\in L}{\mathbb{P}}_{\text{prag}}\left(m|p\right)\sum_{p^{\prime }\in M}{\mathbb{P}}_{\text{prag}}\left(p^{\prime }|m\right)\cdot u\left(p,p^{\prime }\right),$$ (7)

where *u* can be either graded or binary communicative utility. Mirroring our definition of literal communicative cost (Equation 2), we define pragmatic communicative cost as *Cost*_prag_(*L*) = 1 − *I*_prag_(*L*).

#### Results.

**Figure 2.**
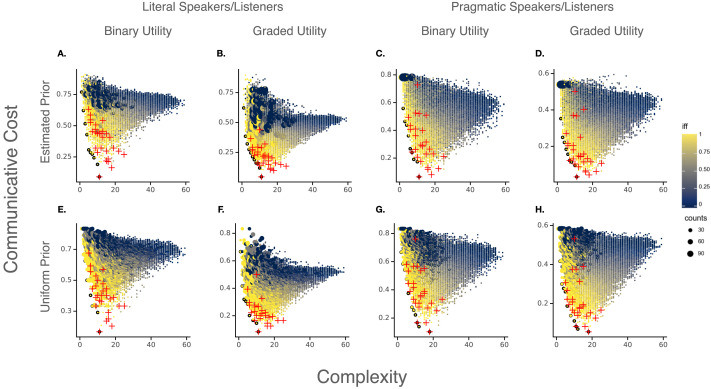
The complexity/communicative cost trade-off for modal languages, evaluated with eight parameter variations for measuring communicative cost. Note that subplot (**B**) is a reproduction of Figure 1. Subplots (**A**–**D**) are obtained with the estimated prior over meanings, while (**E**–**H**) are obtained assuming a uniform prior. (**A/E**) The results obtained for literal speakers and listeners, with respect to a binary utility score for communicative interactions. (**B/F**) Results assuming literal speakers and listeners and a graded communicative utility function. (**C/G**) Results assuming pragmatic speakers and listeners and binary utility. (**D/H**) Results obtained for pragmatic speakers and listeners and graded utility.

For each combination of these three different manipulations—literal vs. pragmatic speakers and listeners, binary vs. graded communicative payoff, and an estimated vs. uniform prior over meanings—we repeated our main analysis, resulting in eight total analyses. The predicted complexity/communicative cost trade-offs are depicted in Figure 2. For all variations just mentioned, each of the three trends from the main results are reproduced: (1) every Pareto-optimal system is perfectly natural (has degree-1.0 IFF satisfaction); (2) a language’s degree of naturalness correlates with simplicity, informativeness, and Pareto-optimality; and (3) the attested modal systems are more Pareto-optimal than the vast majority of hypothetical systems. We report quantitative analyses of modal vocabulary efficiency in Table 7.

**Table 7.** Quantitative analysis of the efficiency of modal languages for different models of communicative informativeness. Each analysis (separated by a light gray line) measures a total sample of 32301 languages. Column 1 (**Prior**): type of assumed communicative need distribution (prior over meanings). Column 2 (**Speaker/Listener**): type of assumed speaker/listener. Column 3 (**Utility**): type of assumed communicative utility function. Column 4 (**Property**): The property measured of a language. Column 5 (**IFF corr.**): Pearson *r* correlation coefficient of the property with naturalness (degree-IFF). Column 6 (**natural**): mean value of the property with respect to the 27 attested languages. Column 7 (**population**): mean value of the property with respect to the total sample of 32301 languages. Column 8 (**nat.** − **pop.**): the difference betweeen a property’s mean value in the attested languages and in the total sample.

Prior	Speaker/Listener	Utility	Property	IFF corr.	natural	population	nat. − pop.
estimated	literal	binary	simplicity	.484	.790	.647	.143
			informativeness	.530	.612	.338	.274
			optimality	.516	.918	.773	.145
		graded	simplicity	.484	.790	.647	.143
			informativeness	.576	.789	.536	.252
			optimality	.537	.937	.776	.160
	pragmatic	binary	simplicity	.484	.790	.647	.143
			informativeness	.428	.685	.463	.222
			optimality	.556	.928	.784	.144
		graded	simplicity	.484	.790	.647	.143
			informativeness	.583	.821	.637	.184
			optimality	.611	.928	.764	.164
uniform	literal	binary	simplicity	.484	.790	.647	.143
			informativeness	.568	.590	.338	.252
			optimality	.542	.932	.780	.152
		graded	simplicity	.484	.790	.647	.143
			informativeness	.618	.772	.536	.236
			optimality	.540	.937	.778	.159
	pragmatic	binary	simplicity	.484	.790	.647	.143
			informativeness	.478	.618	.398	.220
			optimality	.567	.937	.790	.147
		graded	simplicity	.484	.790	.647	.143
			informativeness	.639	.790	.590	.200
			optimality	.687	.935	.745	.190

### Alternative Formalizations of Complexity and Informativeness

The above analyses demonstrated the robustness of our results to internal parameter variations on informativeness. One striking finding is that even a relatively unsophisticated model—with binary utility and a uniform prior—reproduces the overall finding. This raises the question of which aspects of the model are key to producing this pattern. To examine whether our specific formalizations of efficiency are crucial to producing these consistent results, we compare our primary model against alternative theoretical formalizations of both informativeness and complexity.

#### Information Bottleneck Informativeness.

To further contextualize our notion of efficiency within existing frameworks, we also evaluated informativeness as it features in the Information Bottleneck (IB) framework (Zaslavsky et al., 2018), which applies general methods from compression in information theory to efficient communication. Although IB also makes use of an information-theoretic notion of complexity, we here combine its notion of informativeness with our description-length-based complexity measure.^20^

The IB informativeness measure quantifies the relevant information, in bits, that a speaker’s signal conveys about an intended meaning. As part of this measure, we consider the IB communicative cost, which is defined as the KL divergence between a speaker’s probabilistic meaning and an ideal listener’s inferred meaning:

$${\text{Cost}}_{IB}\left(L\right)={𝔼}_{\mathbb{P}\left(p\right),\mathbb{P}\left(m|p\right)}\left[D_{KL}\left[\mathbb{P}\left(p^{\prime }|p\right)\|\mathbb{P}\left(p^{\prime }|m\right)\right]\right]$$ (8)

Note that this measure of communicative cost makes several different assumptions compared to our original expected utility (cost)-based measure: first, it assumes that a (literal) speaker maintains uncertainty over their intended meaning ℙ(*p*′|*p*). Second, it assumes the listener ℙ(*p*′|*m*) is Bayesian. Following Zaslavsky et al. (2021), we implement the probabilistic speaker meanings by considering a similarity-based distribution derived from a rescaled version of our half-credit utility function. Specifically, we define ℙ(*p*′|*p*) ∝ exp(*u*(*p*, *p*′)). In our analyses reported below, we restricted our analyses to need probabilites ℙ(*p*) estimated from the modality corpus (Pyatkin et al., 2021) described in Methods section. The IB informativeness measure is known as the accuracy of the lexicon, defined by *I*(*M*; *P*′) = *I*(*P*; *P*′) − *Cost*_*IB*_(*L*) (for details, see Zaslavsky et al., 2018).

#### Alternative Complexity Measures.

While our primary analysis employed a minimum description length (MDL)-based complexity measure, we also investigated two simpler alternatives for complexity. First, following Regier et al. (2007), we consider the number of distinct forms in a language (i.e., the vocabulary size, which we denote as |*L*|). Second, we consider the entropy of the language’s vocabulary: *H*(*M*) = −∑_*m*∈*L*_ ℙ(*m*) log ℙ(*m*), where ℙ(*m*) = ∑_*p*_ ℙ(*p*)ℙ(*m*|*p*). This latter measure corresponds to the average surprisal, in bits, of modal expressions in the modal language. This quantity is minimized when only one expression is used with nonzero probability, and maximized when all expressions are used with equal probability.^21^

#### Results.

**Figure 3.**
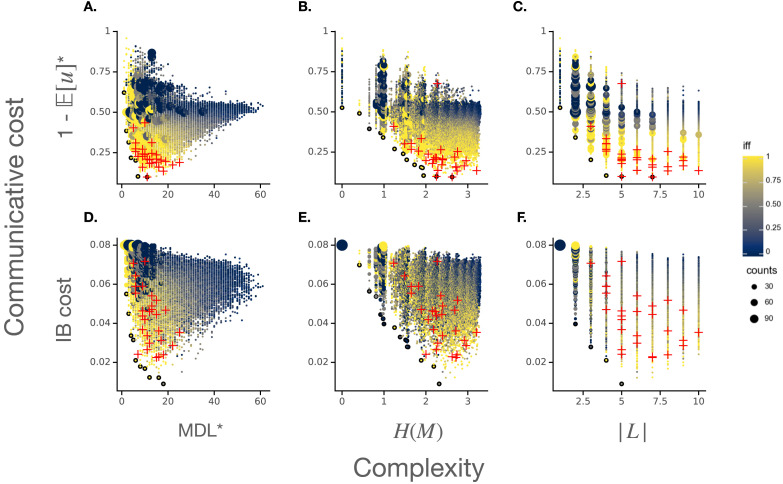
The complexity/communicative cost trade-off for modal languages, evaluated with different ways of measuring complexity and communicative cost. The top row (**A–C**) is evaluated with our main reported measure of communicative cost (1 − 𝔼[*u*], indicated with *), which is based on an expected utility for literal speakers and listeners (Equation 2). The bottom row (**D–F**) evaluates modal languages with respect to the Information Bottleneck (Zaslavsky et al., 2018) communicative cost. The left columns (**A/C**) evaluate the complexity of modal languages with respect to our minimum description length metric (MDL, indicated with *), while the middle and right columns do so with respect to the entropy over modal forms (*H*(*M*)) and the total number of forms (|*L*|), respectively. See the main text for details on these different measures. Note that subplot (**A**) is a reproduction of Figure 1.

For each combination of these three different manipulations—original vs. IB informativeness, and original (MDL-based) complexity vs. vocabulary entropy vs. vocabulary size—we again repeated our analysis, resulting in six additional variants. The predicted complexity/communicative cost trade-offs are depicted in Figure 3. We report quantitative analyses of modal vocabulary efficiency in Table 8.

**Table 8.** Quantitative analysis of the efficiency of modal languages comparing different formalizations of informativeness and complexity. As in Table 7, each analysis (separated by a light gray line) measures a total sample of 32301 languages. Column 1 (**Informativeness**): the informativeness measure considered, either our main reported measure (“original”), based on literal speakers and a half-credit utility function, or an informativeness metric derived from this original measure and the Information Bottleneck (IB) framework. Column 2: (**Complexity**): the complexity measure used, which is either our main reported measure based on a minimum description length in a modal LoT (“MDL”), the entropy of the vocabulary, or simply the size of the vocabulary. Columns 3–7 correspond to Columns 4–8 of Table 7. Note also that the rows 1–3 are rows 4–6 in Table 7.

Informativeness	Complexity	Property	IFF corr.	natural	population	nat. − pop.
original	MDL	simplicity	.484	.789	.647	.142
		informativeness	.576	.789	.536	.252
		optimality	.537	.937	.776	.160
	vocabulary entropy	simplicity	−.006	.306	.286	.020
		informativeness	.576	.789	.536	.252
		optimality	.365	.895	.738	.153
	vocabulary size	simplicity	−.070	.389	.372	.017
		informativeness	.576	.789	.536	.252
		optimality	.123	.847	.714	.132
IB	MDL	simplicity	.484	.789	.647	.142
		informativeness	.594	.055	.023	.033
		optimality	.682	.821	.520	.301
	vocabulary entropy	simplicity	−.006	.306	.286	.020
		informativeness	.594	.037	.021	.016
		optimality	.576	.807	.530	.277
	vocabulary size	simplicity	−.070	.389	.372	.017
		informativeness	.594	.037	.021	.016
		optimality	.540	.772	.506	.266

Under the combination of IB informativeness and MDL complexity, the core efficiency signature is preserved: (1) every Pareto-optimal language is perfectly natural, (2) naturalness correlates with simplicity, informativeness, and Pareto-optimality, and (3) attested modal systems are significantly more Pareto-optimal than the vast majority of hypothetical systems.

The three main results from our original model are not preserved, however, under the two alternative complexity measures. IFF is weakly negatively correlated with both vocabulary entropy and vocabulary size (the high IFF–optimality correlations observed in these conditions are driven by informativeness). Moreover, natural languages are no longer distinguishable from the rest of the population: the mean vocabulary entropy of natural languages is not significantly greater than the full population mean (*t* ≈ .75, *p* ≈ .23), and a parallel analysis for vocabulary size yields a similar result (*t* = .48, *p* ≈ .32). When combined with our original informativeness measure, most, but not all Pareto-optimal languages no longer have degree-1.0 naturalness (<60%); meanwhile, when combined with the IB informativeness measure, only a minority of Pareto languages have degree-1.0 naturalness (<40%). Finally, Figure 3 shows that natural languages appear to cluster nearest to the Pareto frontier when either our original informativeness measure or our MDL-based complexity measure is used.

Taken together, our robustness checks deliver two main upshots. First, despite some variability across parameterizations of informativeness, the major trends and general picture remain stable: the modal typology optimizes for communicative efficiency, and the shared property of all natural language modals—the IFF semantic universal—appears to emerge from this optimization. Second, these core results depend strongly on our original formulation of complexity, suggesting that our modeled representation language for modality encodes an important constraint for capturing the efficiency patterns that distinguish natural languages from hypothetical alternatives.

## DISCUSSION

Let us take stock. We set out to address whether modal typology can be accounted for in terms of pressures for efficient communication. To do this, we defined a complexity measure for modal semantic systems, motivated from the idea that the complexity of a mental representation will be correlated with its shortest description in a Boolean Language of Thought. We also considered several different measures of informativeness, varying our assumptions about how speakers and listeners might successfully communicate about modal meanings. We collected 27 attested modal inventories from 17 different language families, and situated facts about their typological variation in terms of a recently proposed lexical semantic universal, the Independence of Force and Flavor (IFF). We then performed computational experiments to evaluate whether typological variation in the world’s attested modal semantic systems, and the modal semantic universal, can be explained by pressures for communicative efficiency.

We found that communicative efficiency, operationalized in terms of a joint optimization of competing pressures for simplicity and informativeness, explains much of the observed variation in modal semantic typology. Specifically, we found that (1) every Pareto-optimal solution to the simplicity/informativeness trade-off perfectly satisfies the IFF universal; (2) the degree of naturalness for a language, measured in terms of the number of IFF modals it has, is highly correlated with optimality; and (3) the attested modal inventories are more optimal than the vast number of hypothetically possible modal inventories. To ensure the generality of our findings, we systematically varied our models for communicative efficiency, testing a variety of distinct measures of informativeness measures and of complexity. While the main patterns persisted under some combinations of alternative trade-off pressures, the most comprehensive explanations of natural language efficiency emerged with our original formulations of complexity and informativeness.

Pressure for efficient communication, in the simplicity/informativeness trade-off sense, appears to explain important variation in modal typology, at least under some formalizations of this trade-off. It surely does not explain all of it. Importantly, few of the modal systems in our sample were found to perfectly optimal, and many have substantial distance from the Pareto frontier. One reason that these systems are further from the frontier is that they may have high degrees of *synonymy*: languages contain multiple modals that encode the same meaning. Mandarin is one such language, containing ten modals overall: three modals that all ‘mean’ (i.e., have as their minimal LoT formula) strong ∧ epistemic, three modals for strong ∧ ¬epistemic, two modals for weak ∧ epistemic, and two modals for weak ∧ ¬epistemic. Tlingit exhibits a similar degree of synonymy under our analysis: three out of the five lexical items in its modal inventory express weak ∧ epistemic. Such synonymy always hurts the trade-off: it adds complexity without conferring any additional usefulness in informativity. To quantify the impact of synonymy on these deviations from optimality, we also repeated our main analysis after eliminating synonyms from languages and found that natural languages, including Tlingit and Mandarin, are substantially closer to the Pareto frontier after doing so (for details on this analysis, see Appendix A).

This raises a number of interesting possibilities. We suspect that modals that we treat as absolute synonyms are lexical items that differ along additional dimensions of meaning that are simply not captured by our coarse-grained semantic features. Recall that we only consider weak and strong modal force, and three modal flavors. Furthermore, while we assume every language has *some* means of expressing different modal notions, it is not the case that they will have designated, independent lexical items to do so. They may rely on more complex compositional constructions. For example, Urdu/Hindi expresses modality via a specific set of morpho-syntactic constructions (Bhatt et al., 2011). Additionally, Tlingit has few grammatical strategies to express modality, and instead uses various pragmatic strategies to communicate about its modal categories (Cable, 2017).

It is important to acknowledge that there are many factors shaping semantic typology, efficient communication just being one particularly general and powerful one. It will be important in future work to develop methods to adjudicate between alternative explanations, as well as to distinguish between conceptual/cognitive forces shaping semantic variation from cultural/historical/sociological forces. We have focused on the former because semantic universals seem especially likely to arise from such general cognitive pressures.

There are several salient directions for future research. As previously mentioned, a more detailed efficient communication analysis should (i) represent finer-grained distinctions in meaning than the high-level force-flavor dichotomy that we have considered here, (ii) account for the interactions of modality with other domains of meaning (e.g., tense, aspect, evidentiality), and (iii) measure the contribution of morphosyntactic complexity to the overall complexity of modal expressions (Denić & Szymanik, 2024; Carcassi & Sbardolini, 2023; Mollica et al., 2021). In addition, while we have argued (as have many others) that a part of the lexicon is optimized for efficient communication, it remains less clear how languages optimize the simplicity/informativeness trade-off in cultural evolutionary time (though for some recent work in this direction, see Carlsson et al., 2024; Carr et al., 2020; Chaabouni et al., 2021; Imel, 2023; Imel et al., 2023, 2025; Kågebäck et al., 2020). A diachronic analyis of the efficiency of modals (as for example in Zaslavsky et al., 2022) will also be important for evaluating whether natural language modal systems actively evolve under pressure for efficiency.

Altogether, our empirical results suggest that efficient communication succesfully explains significant variation in modal typology. More generally, our findings lend support to the idea that the humanly-possible languages are the outcomes of competing constraints that arise from representing the environment and coordinating on shared goals. In addition, by explaining how greater optimality results in higher degrees of naturalness, our account is compatible with a view that semantic universals correspond to soft rather than hard constraints. This leaves room for the attractive idea that linguistic ‘universals’ can be observed as robust, statistical tendencies, rather than absolute restrictions (Evans & Levinson, 2009). Here, we have used an efficiency-based analysis to explain why languages may satisfy these tendencies to varying degrees: modal vocabularies are subject to competing pressures for cognitive simplicity and informative communication.

## ACKNOWLEDGMENTS

We are grateful to Toshi Ogihara, Jakub Szymanik, Wataru Uegaki, Noga Zaslavsky, and audiences at Amsterdam, Austin, Davis, Irvine, and MIT for helpful discussion. We also thank James Engels, Ella Hannon, Anne Mucha, Wataru Uegaki, Fred Whibley, Minghe Zhang, Zoe Fang and Ian Chandra Lim for their work on contributing primary modals data to the modal typology database, described in Typological Data section.

## AUTHOR CONTRIBUTIONS

N.I.: Conceptualization; Formal analysis; Investigation; Methodology; Project administration; Software; Validation; Visualization; Writing – original draft; Writing – review & editing. Q.G.: Data curation; Investigation; Validation; Writing – review & editing. S.S.-T.: Conceptualization; Investigation; Methodology; Project administration; Software; Supervision; Validation; Writing – original draft; Writing – review & editing.

## DATA AVAILABILITY STATEMENT

The modal typological data used in this work can be found at https://clmbr.shane.st/modal-typology/. The code for reproducing the results can be found at https://github.com/nathimel/modals-effcomm.

## Notes

^1^ Typical implementations determine the flavor as the product of two further parameters: a modal base and an ordering source. We set aside this distinction for present purposes.^2^ We will discuss modals that specify neither force nor flavor in the next section.^3^ These are examples (5c) and (5e) from Rullmann et al. (2008): 321. See their footnote 5 on p. 320 for the abbreviations.^4^ In addition to the particular studies already mentioned, see Cable (2017) and Matthewson (2013) for more examples of the application of these methods.^5^ We intend ‘judged felicitous’ to also include the case where such sentences are produced naturally in elicitation tasks, as well as when such sentences are found in naturally-occuring contexts which have a clear force-flavor pair.^6^ Here is the formulation in Nauze (2008, p. 222): “Modal elements can only have more than one meaning along a unique axis of the semantic space: they either vary on the horizontal axis and thus are polyfunctional in the original sense of expressing different types of modality or they vary on the vertical axis and can express possibility and necessity, but they cannot vary on both axes.”^7^ There are also apparently bouletic uses of *ivək*, but Močnik and Abramovitz (2019) argue that this flavor does not come from *ivək* alone but from interaction with material in the embedded clause.^8^ One sense in which the formulation can be seen as ‘theory-netural’: Kratzer (1981) builds in independence by treating force as lexically encoded and flavor as contextually determined. The present level of analysis does not commit to any positive view on which components are lexically specified and which are not. Thanks to Wataru Uegaki (p.c.) for discussion here.^9^ We note that Imel and Steinert-Threlkeld (2022) and Imel (2022) previously applied a similar analysis to explore whether the SAV and DL-SAV universals can be accounted for in terms of pressure for efficient communication. We focus on the IFF universal here, because it appears to more accurately capture the empirical landscape of modal typology.^10^ Defining a language to be a multi-set of modals allows for synonymy.^11^ For more on circumstantial and other flavors, see Matthewson (2016).^12^ The full details of this algorithm, including pseudocode, can be found in Appendix A of Steinert-Threlkeld (2021). In addition to the three mutations applied to entire languages in that algorithm (namely, add, remove, and interchange), we use two mutations that apply at the level of individual lexical items: add_point and remove_point. The add_point mutation adds a random force/flavor pair to the meaning of a randomly chosen modal expression within a language. Similarly, the remove_point mutation removes a force-flavor pair from a modal.^13^ In our meaning space with two forces and three flavors, there are six meaning points, yielding 2^6^ − 1 = 63 possible modals. For a fixed vocabulary size of 10 modals (allowing for synonymy), there are $\left(\begin{array}{c} k+n-1\\ k \end{array}\right)=\left(\begin{array}{c} 10+63-1\\ 10 \end{array}\right)$ > 5.3 × 10^11^ possible languages.^14^ For robustness, we also computed optimality scores using the Manhattan distance. Details of this analysis can be found in Appendix B.^15^ We note that informativeness exhibits a slightly higher linear correlation with naturalness than optimality here, although in our robustness checks in the following section, this is not always the case. More importantly, such a difference does not imply naturalness optimizes for only one dimension (informativeness), as optimality is a non-linear composite measure of both simplicity and informativeness, which can attenuate its linear correlation even if the underlying pressure is the full, multi-dimensional trade-off.^16^ A one-sided *t*-test revealed that natural languages had scores significantly greater than the population mean *p* ≈ 0, for each of these measures. We omit the exact *t* statistic for each measure for brevity and ease of reading.^17^ Here we describe the *S*_1_ speaker and *L*_1_ listener, which index the ‘first level’ of recursive reasoning, but in general the RSA framework can consider pragmatic reasoning of agents up to arbitrary recursion depth. For example, an *S*_2_ speaker can condition their decisions based on how a pragmatic listener would interpret them, and so on.^18^ For example, if the literal listener never interprets English *may* as expressing (strong, circumstantial), then ℙ((strong, circumstantial)|may) = 0 and *U*_prag_(may, (strong, circumstantial)) = −∞, i.e., the pragmatic speaker has no incenctive to use *may* to express this force-flavor pair. Meanwhile, if the literal listener always interprets English *might* as expressing (weak, epistemic), then ℙ((weak, epistemic)|might) = 1.0 and so *U*_prag_(might, (weak, epistemic)) = 0.0, i.e., saying *might* will maximize the pragmatic speaker’s utility.^19^ This is modeled by the softmax function in Equation 4, which normalizes the utility score of each modal expression to the probability of uttering the expression. The temperature parameter *α* in this function controls the ‘sharpness’ of the resulting distribution. When *α* → ∞, the speaker’s choice of expression depends only on its utility; when *α* = 0, the speaker chooses randomly (and hence will typically be much worse for communication than a literal speaker). In our experiment, we let *α* = 1.0, which reflects the assumption that speakers are neither perfectly rational nor completely incompetent.^20^ We thank an anonymous reviewer for suggesting this specific analysis.^21^ We thank an anonymous reviewer for suggesting these measures of complexity for comparison as well.

## Figures and Tables

**Figure A1.**
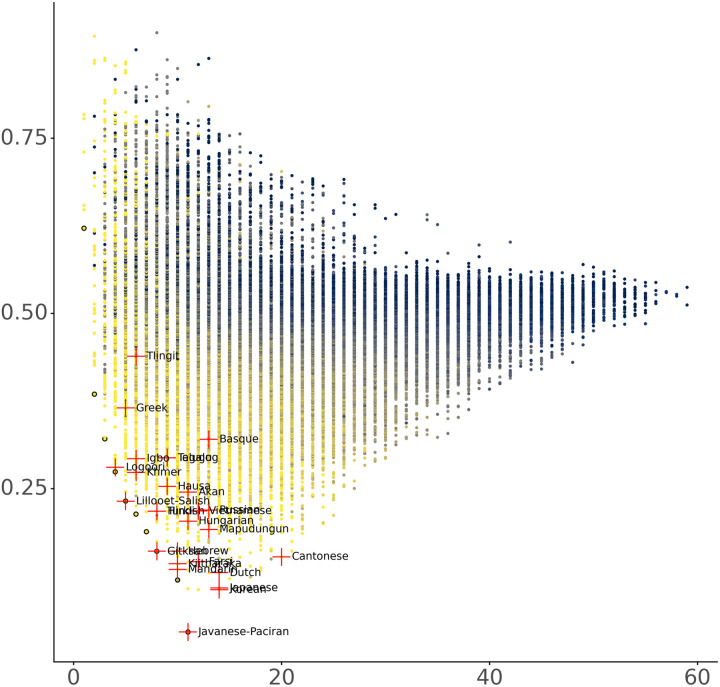
The complexity/communicative cost trade-off for modal languages, removing any synonyms. Compare to Figure 1.

## APPENDIX A: MAIN ANALYSIS WITHOUT SYNONYMY

To investigate the potential influence of synonymy on language optimality, particularly for those natural languages previously observed to deviate from the Pareto frontier, we re-ran our main analysis. In this modified analysis, we specifically prohibited the inclusion of synonymous forms in any language, whether they were attested natural languages loaded from the corpus or generated hypothetical languages. This was achieved by retaining only one expression for each unique set of force-flavor pairs; if multiple expressions conveyed the exact same meaning, they were collapsed into a single representative lexical item. This “de-synonymized” approach allowed us to test the conjecture that the presence of synonymy always makes a language more inefficient, by incurring greater complexity for the same level of informativeness achieved.

As shown in Table A1 and Figure A1, our main findings regarding the greater optimality of natural languages were strengthened under this analysis. Languages such as Tlingit and Mandarin, which appeared further from the frontier in our original analysis, achieved higher optimality in this synonymy-free analysis. For example, omitting two additional expressions for expressing weak ∧ epistemic lowered the complexity of Tlingit from ten to six atoms, and consequently increased optimality from .538 to .949. In Mandarin, eliminating synonymy reduced complexity from 25 to 10 atoms, raising its optimality from .650 to .992. This outcome supports the claim that the presence of synonymy in natural languages introduces additional complexity that pushes them away from the efficient frontier.

**Table A1.** Mean simplicity, informativeness, and Pareto optimality for attested natural languages vs. hypothetical languages. Results are a replication of the main analysis reported (compare to Table 6), while removing any synonyms from languages (natural or hypothetical).

language	*N*	simplicity	informativeness	optimality
mean natural	27	.830	.784	.959
mean population	32301	.647	.536	.776

## APPENDIX B: OPTIMALITY VIA MANHATTAN DISTANCE

In the main text, we define a language’s optimality based on its minimum Euclidean distance (L2 norm) to the Pareto frontier. To ensure further robustness of our findings, we here evaluate optimality using an alternative measure: Manhattan distance (L1 norm). Manhattan distance calculates the sum of the absolute deviations along each objective axis, which is also a relevant metric for assessing proximity in a multi-objective setting. Tables B1 and B2 demonstrate that our core conclusions remain consistent when optimality is measured using Manhattan distance.

**Table B1.** Mean IFF correlation, natural language mean (natural), population mean (population), and the difference between natural and population means (nat. − pop.) for L2 (Euclidean) and L1 (Manhattan) optimality across different model variants. Compare to Table 7.

Prior	Speaker/Listener	Utility	Optimality	IFF corr.	natural	population	nat. − pop.
estimated	literal	binary	L2	.516	.918	.773	.145
			L1	.515	.939	.772	.167
		graded	L2	.537	.937	.776	.160
			L1	.516	.954	.771	.183
	pragmatic	binary	L2	.556	.928	.784	.144
			L1	.569	.949	.774	.175
		graded	L2	.611	.928	.764	.164
			L1	.678	.944	.727	.217
uniform	literal	binary	L2	.542	.932	.780	.152
			L1	.588	.954	.768	.186
		graded	L2	.540	.937	.778	.159
			L1	.517	.952	.773	.179
	pragmatic	binary	L2	.567	.937	.790	.147
			L1	.586	.955	.778	.177
		graded	L2	.687	.935	.745	.190
			L1	.745	.941	.682	.259

**Table B2.** Comparison of L2 (Euclidean) and L1 (Manhattan) optimality results across different informativeness and complexity formalizations. Compare to Table 8.

Informativeness	Complexity	Optimality	IFF corr.	natural	population	nat. − pop.
original	MDL	L2	.537	.937	.776	.160
		L1	.516	.954	.771	.183
	vocabulary entropy	L2	.365	.895	.738	.153
		L1	.366	.905	.655	.250
	vocabulary size	L2	.123	.847	.714	.132
		L1	.035	.876	.660	.216
IB	MDL	L2	.682	.821	.520	.301
		L1	.688	.796	.415	.381
	vocabulary entropy	L2	.576	.807	.530	.277
		L1	.502	.762	.426	.336
	vocabulary size	L2	.540	.772	.506	.266
		L1	.407	.756	.380	.376
